# Intravenous Thiamine-Induced Thrombocytopenia in a Patient With Chronic Adrenal Insufficiency

**DOI:** 10.14740/jmc5250

**Published:** 2026-01-04

**Authors:** Mohammed Ayed Alanazi

**Affiliations:** Department of Medicine, College of Medicine, Majmaah University, Al-Majmaah, Saudi Arabia. Email: maalanazi@mu.edu.sa

**Keywords:** Thiamine, Thrombocytopenia, Adverse drug reaction, Vitamin B1, Drug-induced thrombocytopenia

## Abstract

Thiamine (vitamin B1) is generally considered safe, with rare adverse effects, including anaphylaxis. Although thrombocytopenia related to thiamine deficiency is known to improve with supplementation, thrombocytopenia occurring after thiamine administration has not been well described. We report the case of a 63-year-old female with adrenal insufficiency and malnutrition who developed severe thrombocytopenia shortly after initiation of intravenous thiamine for nutritional support. Platelet counts declined rapidly during therapy and recovered completely following thiamine discontinuation, with no alternative etiology identified after systematic evaluation. Drug-induced immune thrombocytopenia was suspected, with Naranjo score of 6 indicating a probable adverse drug reaction; the temporal relationship and clinical course were consistent with this diagnosis. No alternative causes were identified. This case highlights a rare and previously undocumented association between thiamine therapy and thrombocytopenia. Clinically, this report demonstrates that even medications generally regarded as safe may, in rare cases, lead to serious hematological adverse effects, underscoring the importance of reporting such events to increase clinical awareness of this uncommon but potentially severe reaction. Clinicians should maintain a high index of suspicion for drug-induced thrombocytopenia in patients receiving intravenous thiamine and consider platelet monitoring in high-risk or critically ill patients.

## Introduction

Thiamine (vitamin B1) is an essential water-soluble vitamin crucial for carbohydrate metabolism and neurological function. Clinical use ranges from routine supplementation to high-dose intravenous (IV) therapy for Wernicke’s encephalopathy. Common adverse effects are rare and mild, including gastrointestinal symptoms and injection site reactions. Severe reactions include anaphylaxis, occurring in approximately 1:1,000 to 1:10,000 doses [1, 2]. Other rare effects include pruritus, urticaria, and weakness. Notably, hematologic adverse effects have not been documented in the thiamine safety literature.

Drug-induced immune thrombocytopenia (DITP) affects approximately 10 per 1 million patients annually, with over 300 medications implicated [3]. Importantly, thiamine-deficiency-responsive thrombocytopenia exists where supplementation improves platelet counts in deficiency states [4, 5], representing the opposite phenomenon. To our knowledge, this represents the first reported case of thiamine-induced thrombocytopenia as an adverse drug reaction.

## Case Report

A 63-year-old female with a past medical history of adrenal insufficiency secondary to tuberculosis infection (15 years prior) and controlled hypertension presented to the emergency department. The patient resided in a rural area and had been on long-term maintenance therapy with prednisone (10 mg morning, 5 mg evening) and lisinopril 5 mg daily. Five days before the presentation, she ran out of her medications and was unable to obtain refills.

### Presenting complaints and initial presentation

The patient presented with a chief complaint of diffuse abdominal pain (generalized distribution, moderate intensity 5/10, non-radiating, non-colicky) associated with confusion, nausea, and multiple episodes of vomiting over the preceding 2 days. Upon further questioning, she reported frequent hypoglycemic episodes during the 3 days prior to admission, manifesting as diaphoresis, tremors, and palpitations with documented home blood glucose readings as low as 42 mg/dL. The patient denied fever, diarrhea, hematochezia, melena, chest pain, or dyspnea.

### Initial assessment and emergency department course

On arrival at the emergency department, the patient appeared confused and lethargic with an altered mental status. Initial vital signs revealed hypotension (blood pressure 95/65 mm Hg), with other vital signs within normal limits (pulse rate 82 beat per minute (bpm), respiratory rate 18/min, temperature 36.9 °C, oxygen saturation 97% on room air). Given the history of medication discontinuation, clinical presentation, and hypotension, a presumptive diagnosis of adrenal crisis was made. The patient received aggressive IV fluid resuscitation with normal saline and immediate administration of IV hydrocortisone. She was subsequently admitted to the Internal Medicine service for further management.

Over the initial 48 - 72 h of hospitalization, the patient showed dramatic improvement in mental status and hemodynamic stability with stress-dose corticosteroid therapy (IV hydrocortisone 100 mg bolus followed by 50 mg four times daily) and supportive care. Physical examination revealed a cachectic, malnourished appearance with a body mass index of 16.9 kg/m^2^. She was conscious, alert, and oriented to time, place, and person. Vital signs stabilized (blood pressure 135/88 mm Hg, pulse 78 bpm, respiratory rate 22/min, oxygen saturation 96% on room air). Cardiovascular examination showed regular rate and rhythm without murmurs. Respiratory examination was clear bilaterally. Abdominal examination revealed no distension, organomegaly, or peritoneal signs. Importantly, there were no signs of bleeding, including petechiae, purpura, bruises, or bleeding from any orifices. The remainder of the physical examination was unremarkable.

Given the patient’s poor nutritional status, marked weight loss, and poor oral intake, the medical team decided to initiate nutritional support. A nasogastric tube was inserted after recovery from the presumed adrenal crisis, and enteral nutrition was started with liquid formula (Ensure^®^) four times daily. The patient was transitioned from IV hydrocortisone to her home oral prednisone regimen (10 mg morning, 5 mg evening). Lisinopril remained held during hospitalization.

On hospital day 4, recognizing the risk of refeeding syndrome and thiamine deficiency in this malnourished patient, the team decided to administer prophylactic thiamine supplementation. IV thiamine 100 mg diluted in 100 mL of 5% dextrose in 0.9% normal saline, was initiated and administered once daily via slow infusion over 30 min. Baseline complete blood count obtained on the day of thiamine initiation (day 0) showed normal hematologic parameters, including a platelet count of 225 × 10^9^/L (reference range 150 - 450 × 10^9^/L) (Table 1).

**Table 1:** Baseline Laboratory Values (Day 0)

Parameter	Value	Reference range
Hemoglobin	13.3 g/dL	12.5 - 16.5 g/dL
WBC	7.5 × 10^9^/L	4.0 - 11.0 × 10^9^/L
Platelets	225 × 10^9^/L	150 - 450 × 10^9^/L
MCV	96 fL	78 - 98 fL
Reticulocytes	1.2%	0.5-2.5%

WBC: white blood cell; MCV: mean corpuscular volume.

### Thrombocytopenia development and clinical recognition

On day 1 after thiamine initiation, routine laboratory monitoring showed an unexpected decline in platelet count to 150 × 10^9^/L (33% decrease from baseline). Initially attributed to laboratory variation or dilutional effect from fluid resuscitation, repeat testing on day 2 demonstrated further decline to 110 × 10^9^/L (51% decrease). Thrombocytopenia was graded according to the Common Terminology Criteria for Adverse Events (CTCAE), which classifies adverse events based on severity. By day 3, platelet count had dropped to 86 × 10^9^/L, meeting CTCAE grade 1 thrombocytopenia criteria. Repeat testing with both citrate anticoagulant and peripheral blood smear examination definitively confirmed true thrombocytopenia and excluded pseudothrombocytopenia (EDTA-induced platelet clumping).

The progressive decline continued: day 4 platelet count 63 × 10^9^/L (grade 2 CTCAE), and day 5 reaching nadir of 48 × 10^9^/L (grade 3 CTCAE, 79% reduction from baseline). According to CTCAE version 5.0, grade 3 thrombocytopenia is defined as a severe adverse event characterized by a platelet count < 50 × 10^9^/L, often requiring clinical intervention. Throughout this period, comprehensive hematologic workup was initiated, and both Internal Medicine physicians and Hematology consultants evaluated the patient. Notably, despite severe thrombocytopenia, the patient remained asymptomatic without bleeding manifestations, petechiae, purpura, mucosal bleeding, or other hemorrhagic complications. She was placed under close observation with serial examinations (Table 2).

**Table 2:** Platelet Count Timeline and Common Terminology Criteria for Adverse Events (CTCAE) Grading

Day	Intervention	Platelet count (× 10^9^/L)	% Change	CTCAE grade
0 (baseline)	Before thiamine (day of initiation)	225	0	0
1	Day 1 after thiamine started	150	-33%	0
2	Day 2 - thiamine continued	110	-51%	0
3	Day 3 - thiamine continued	86	-62%	Grade 1
4	Day 4 - thiamine continued	63	-72%	Grade 2
5	Day 5 - thiamine continued	48	-79%	Grade 3
6	Day 6 - thiamine stopped	65	+35%	Grade 2
7	Day 7 - post-cessation	88	+83%	Grade 2
8	Day 8 - post-cessation	132	+175%	Grade 1
14	Day 14 - follow-up (2 weeks)	265	Complete recovery	0

After systematic exclusion of common causes of thrombocytopenia (Table 3), comprehensive medication chart review revealed that thiamine was the only new medication introduced, with temporal correlation between thiamine initiation and platelet count decline beginning on day 1. Peripheral smear showed clear thrombocytopenia without clumping and a few giant platelet forms. No concurrent medications were introduced except thiamine. Lisinopril was held during admission.

**Table 3:** Systematic Exclusion of Alternative Causes

Condition	Investigations	Results	Conclusion
Sepsis	Vitals, CRP, blood cultures	BP 135/88, CRP 0.88 mg/dL, cultures negative	Excluded
DIC	PT, aPTT, INR, fibrinogen, D-dimer	PT 10 s, aPTT 26 s, INR 0.9, Fib 360 mg/dL, D-dimer 0.50	Excluded
HIT	Heparin exposure, 4T score	No heparin exposure, 4T score = 0	Excluded
Autoimmune	ANA, Coombs, ESR	ANA negative, Coombs negative, ESR 18	Excluded
TTP/HUS	LDH, haptoglobin, schistocytes	LDH 160 IU/L, haptoglobin 52 mg/dL, no schistocytes	Excluded
Bone marrow	CBC, peripheral smear	Isolated thrombocytopenia, normal morphology	Excluded
HIV infection, Hep C, and Hep B	HIV Ag/Ab test, anti-HCV antibody, HBsAg, anti-HBc, and anti-HBs	Negative, negative, negative, negative, and positive	Excluded and vaccinated Hep B
Liver function test	AST, ALT, alkaline phosphatase, GGT, total bilirubin	34 U/L, 38 U/L, 56 U/L, 22U/L, 1.1 mg/dL	Normal test
Renal function and electrolytes	Creatinine, urea, Na, K, Cl, Mg, Ca	1.1 mg/dL, 22 mg/dL, 134 mmol/L, 3.7 mmol/L, 99 mmol/L, 1.9 mg/dL, 9.3 mg/dL	Borderline test
Vitamin B12 and folate	Serum values of vitamin B12 and folate	369 pmol/L, 14 ng/mL	Normal
RBC count	RBC count	5.1 × 10^6^/µL	Normal

DIC: disseminated intravascular coagulation; HIT: heparin-induced thrombocytopenia; TTP: thrombotic thrombocytopenic purpura; HUS: hemolytic uremic syndrome; HIV: human immunodeficiency virus; Hep C: hepatitis C virus; Hep B: hepatitis B virus; RBC: red blood cell; CRP: C-reactive protein; PT: prothrombin time; aPTT: activated partial thromboplastin time; INR: international normalized ratio; ANA: antinuclear antibody; ESR: erythrocyte sedimentation rate; LDH: lactate dehydrogenase; CBC: complete blood count; HIV Ag/Ab test: human immunodeficiency virus antigen/antibody test; anti-HCV antibody: hepatitis C virus antibody; HBsAg: hepatitis B surface antigen; anti-HBc: hepatitis B core antibody; anti-HBs: hepatitis B surface antibody; AST: aspartate aminotransferase; ALT: alanine aminotransferase; GGT: gamma-glutamyl transferase.

Given the compelling temporal relationship, absence of alternative explanations, and progressive severity despite supportive care, DITP secondary to thiamine was suspected. IV thiamine was immediately discontinued on day 5.

Within 24 h of thiamine discontinuation (day 6), platelet count showed initial recovery to 65 × 10^9^/L (35% increase from nadir). Continued improvement was documented: day 7 (88 × 10^9^/L), day 8 (132 × 10^9^/L). The patient remained clinically stable throughout recovery with no bleeding complications. She was discharged home on day 10 with close hematology follow-up arranged. At 2-week follow-up (day 14), the patient reported no bleeding symptoms, physical examination was unremarkable, and repeat complete blood count showed complete platelet recovery to 265 × 10^9^/L (within normal range). Long-term follow-up at 3 months confirmed sustained normal platelet counts with no recurrence, and the patient was advised to avoid all forms of thiamine supplementation in the future.

## Discussion

This case represents the first documented report of thiamine-induced thrombocytopenia as an adverse drug reaction in the medical literature. An extensive search of major databases revealed no prior cases where thiamine administration caused a decline in platelet count [2, 6]. This is particularly remarkable given the widespread use of thiamine in clinical practice. Importantly, this phenomenon must be distinguished from thiamine-deficiency-responsive thrombocytopenia, in which supplementation improves platelet counts in deficiency states. Only two to three cases of thiamine-responsive thrombocytopenia have been reported [4, 5], in which patients with profound deficiency developed thrombocytopenia that resolved with thiamine replacement, which is the opposite of our patient. In deficiency-responsive cases, thiamine corrects underlying metabolic disturbances affecting megakaryopoiesis [4], whereas in our case, thiamine may have triggered immune-mediated platelet destruction.

This novel adverse drug reaction has significant implications for pharmacovigilance and patient safety [7].

Causality assessment strongly supports a probable causal relationship. Using the validated Naranjo Adverse Drug Reaction Probability Scale [8], our case achieved a score of six points, classified as “probable” adverse drug reaction: temporal relationship (+ 2), improvement after discontinuation (+ 1), exclusion of alternatives (+ 2), and objective evidence (+ 1). Additionally, the case satisfies three of four George criteria [3, 9]: 1) temporal relationship with therapy; 2) complete recovery after discontinuation; and 3) systematic exclusion of other causes. The fourth criterion - rechallenge - was not performed because it was ethically inappropriate given grade 3 CTCAE severity [1].

Current DITP guidelines [10] support avoiding rechallenge when causality can be reasonably established, particularly in severe cases. The compelling temporal sequence, including platelet decline within 24 h, progressive deterioration during exposure, and complete recovery after cessation, provides robust evidence even without rechallenge [11, 12]. The dose-duration relationship, demonstrated by a progressive decline over 5 consecutive days, further strengthens causal inference [7]. The pathophysiologic mechanisms remain speculative, but established DITP frameworks [11, 13] provide plausible explanations. The most likely mechanism involves hapten-dependent antibody formation [11], where thiamine’s reactive functional groups bind platelet membrane glycoproteins (GPIIb/IIIa or GPIb/IX), creating immunogenic complexes that trigger drug-specific antibody production. Upon continued exposure, these antibodies mark thiamine-coated platelets for Fc receptor-mediated phagocytosis.

Alternatively, a drug-dependent platelet antibody mechanism of the quinine-type may be involved [12]. In this mechanism, the drug enhances the binding affinity of pre-existing antibodies to platelet antigens without covalent attachment, which allows targeted antibody testing to have supportive diagnostic value [9]. This mechanism could explain the rapid onset of thrombocytopenia within 24 h, as circulating antibodies would already be present. The antibody testing would be helpful in this type of mechanism, but it was not available in our clinical setting. However, the quinine-type drug-dependent antibody mechanism is a recognized pathway in DITP; prior exposure to thiamine in this patient could not be confirmed, which limits reliance on this mechanism. Therefore, the diagnosis was based on clinical criteria, including temporal association, exclusion of alternative causes, and platelet recovery after thiamine discontinuation.

A third possibility involves immune complex formation [11], where thiamine-antibody complexes bind platelets as “innocent bystanders,” triggering complement activation and clearance. The progressive decline suggests cumulative immune-mediated injury rather than direct toxicity. Complement-mediated destruction may contribute, as documented in quinidine and vancomycin DITP [13]. Unfortunately, specialized antibody testing was unavailable, precluding mechanistic confirmation. Future studies should employ flow cytometry to detect drug-dependent antibodies and characterize target glycoproteins [10].

Several limitations must be acknowledged [14]. First, definitive causation cannot be established from single observations without rechallenge or additional cases. Second, specialized antibody testing was unavailable, which would have provided mechanistic confirmation [10]. Third, occult viral infections were not definitively excluded, though the immediate temporal relationship and rapid recovery are atypical for viral thrombocytopenia. Fourth, coincidental immune thrombocytopenia (ITP), while improbable, cannot be dismissed; however, spontaneous recovery without ITP-specific treatments [15, 16] argues against this. Fifth, bone marrow examination was not performed, though rapid recovery and isolated thrombocytopenia make marrow failure unlikely. Despite these limitations, the temporal association strength, systematic alternative exclusion, grade 3 severity, and complete recovery provide robust support for probable causality [8, 11].

This case has substantial clinical implications for thiamine administration practices [6, 17]. While routine platelet monitoring is not currently recommended given thiamine’s excellent safety profile, monitoring may be prudent in high-risk scenarios: 1) high-dose IV thiamine (≥ 100 mg/day) for multiple consecutive days; 2) patients with prior DITP history; 3) pre-existing autoimmune disorders; and 4) malnourished or critically ill patients on multiple medications [10, 12, 16].

The author proposes risk-stratified monitoring: standard-risk patients (oral or single-dose parenteral) require no routine monitoring; intermediate-risk patients (high-dose IV) should have baseline CBC with repeat testing if bleeding develops; high-risk patients warrant daily platelet counts for 5 - 7 days, representing peak risk based on observed DITP patterns [10, 11, 13]. If thrombocytopenia develops, immediately discontinue thiamine, exclude alternative causes, and provide supportive care with transfusion if platelets fall below 10 × 10^9^/L [15, 16]. Affected patients must avoid future thiamine supplementation with prominent documentation in medical records [10]. The excellent prognosis with complete 2-week recovery is reassuring. This case contributes important pharmacovigilance data and highlights the need for continued vigilance even with drugs having excellent safety profiles [1, 14].

### Conclusions

This represents the first documented case of thiamine-induced thrombocytopenia as an adverse drug reaction. Despite inherent single case report limitations, the strong temporal association (Naranjo score 6), systematic exclusion of alternatives, grade 3 CTCAE severity, and complete recovery support probable causality. Clinicians should maintain awareness of this rare but serious adverse effect, particularly with high-dose IV thiamine therapy. The excellent prognosis following discontinuation is reassuring. This case contributes important pharmacovigilance data for a widely used medication and supports consideration of platelet monitoring in high-risk scenarios.

## Data Availability

Any inquiries regarding supporting data availability of this study should be directed to the corresponding author.
